# Painful ejaculation induced by venlafaxine: a case report

**DOI:** 10.1192/j.eurpsy.2022.2071

**Published:** 2022-09-01

**Authors:** F. Kulacaoglu

**Affiliations:** Istanbul Bakirkoy Prof Dr. Mazhar Osman Research and Training hospital for mental health and neurological diseases, Psychiatry, istanbul, Turkey

**Keywords:** painul ejaculation, sexual medicine, antidepressant treatment, venlafaxine

## Abstract

**Introduction:**

Sexual dysfunction is a quite common side effect of antidepressant treatment. Sexual side effects may affect the person’s adherence to treatment, quality of life, and relations. Premature ejaculation is rarely seen as an adverse effect of antidepressant drugs.

**Objectives:**

We aimed to present a clinical case of a 53-year-old man who developed painful ejaculation with the use of venlafaxine.

**Methods:**

We made a narrative literature search in Pubmed and Google scholar with the terms of painful ejaculation induced by venlafaxine and antidepressant treatment.

**Results:**

A 53-year-old man was admitted to the psychiatric outpatient unit with symptoms of anhedonia, decreased sleep, decreased self-esteem for the last month. The patient was diagnosed with depression and he started to take 37,5 mg venlafaxine per day. After one month, when venlafaxine dose was increased to 75mg and the patient started to complain of painful ejaculation. The pain continued from the beginning to the end of the ejaculation. The pain increased more when the venlafaxine dose increased to 150mg per day. The patient was consulted at the urology clinic. The urological examination, laboratory tests (direct microscopic examination of the urethral discharge and urethral culture), and serum prostate-specific antigen levels were normal. No pathology was found in uroflowmetry and ultrasonography of the urinary system. The dose of venlafaxine decreased and the patient started to take 20 mg of fluoxetine per day. His symptoms disappeared after venlafaxine was discontinued.

**Conclusions:**

To literature, this is the second presentation of painful ejaculation observed during the use of venlafaxine.

**Disclosure:**

No significant relationships.

